# Rise in DPA Following SDA-Rich Dietary Echium Oil Less Effective in Affording Anti-Arrhythmic Actions Compared to High DHA Levels Achieved with Fish Oil in Sprague-Dawley Rats

**DOI:** 10.3390/nu8010014

**Published:** 2016-01-04

**Authors:** Mahinda Y. Abeywardena, Michael Adams, Julie Dallimore, Soressa M. Kitessa

**Affiliations:** Commonwealth Scientific and Industrial Research Organisation (CSIRO) Food & Nutrition, Kintore Ave, Adelaide SA 5000, Australia; Michael.Adams@csiro.au (M.A.); Julie.Dallimore@csiro.au (J.D.); Soressa.Kitessa@csiro.au (S.M.K.)

**Keywords:** *n*-3 fatty acids, fish oil, Echium oil, stearidonic acid, docosapentaenoic acid, docosahexaenoic acid, eicosapentaenoic acid, cardiac arrhythmia, rat

## Abstract

Stearidonic acid (SDA; C18:4*n*-3) has been suggested as an alternative to fish oil (FO) for delivering health benefits of C ≥ 20 long-chain *n*-3 polyunsaturated fatty acids (LC *n*-3 PUFA). Echium oil (EO) represents a non-genetically-modified source of SDA available commercially. This study compared EO and FO in relation to alterations in plasma and tissue fatty acids, and for their ability to afford protection against ischemia-induced cardiac arrhythmia and ventricular fibrillation (VF). Rats were fed (12 weeks) diets supplemented with either EO or FO at three dose levels (1, 3 and 5% *w*/*w*; *n* = 18 per group). EO failed to influence C22:6*n*-3 (DHA) but increased C22:5*n*-3 (DPA) in tissues dose-dependently, especially in heart tissue. Conversely, DHA in hearts of FO rats showed dose-related elevation; 14.8%–24.1% of total fatty acids. Kidney showed resistance for incorporation of LC *n*-3 PUFA. Overall, FO provided greater cardioprotection than EO. At the highest dose level, FO rats displayed lower (*p* < 0.05) episodes of VF% (29% *vs.* 73%) and duration (22.7 ± 12.0 *vs.* 75.8 ± 17.1 s) than the EO group but at 3% EO was comparable to FO. We conclude that there is no endogenous conversion of SDA to DHA, and that DPA may be associated with limited cardiac benefit.

## 1. Introduction

The influence of dietary fats on the pathogenesis of coronary heart disease, congestive heart failure as well as vulnerability to cardiac arrhythmias and sudden cardiac death has been well documented [1,2]. In this regard, both the “type” and the “amount” of dietary oils and fats have been identified as important determinants [3,4,5]. For example, a considerable body of supporting evidence shows that long chain (C ≥ 20) *n*-3 polyunsaturated fatty acids (LC *n*-3 PUFA) derived from marine sources (seafood, fish and microalgae) are particularly effective in affording cardiovascular protection [6,7] although more recent analyses have reported inconsistent outcomes [8]. In similar vein, a review of recent clinical trials (2007–2013 period) showed a lack of clear benefit of fish oil supplements although high dietary intake of fish was associated with lower incidence of sudden cardiac death, congestive heart failure, myocardial infarction and stroke [9]. Among the *n*-3 PUFA, the two major LC- *n*-3 PUFA are eicosapentaenoic acid (EPA, C20:5*n*-3) and docosahexaenoic acid (DHA, C22:6*n*-3).

A number of studies [10,11] have reported certain positive cardiovascular health outcomes from consumption of α-linolenic acid (ALA, C18:3*n*-3), an essential *n*-3 PUFA widely available from plant-based food sources including certain seed oils (e.g., flax, canola, perilla, chia, walnut, *etc.*). The primary mechanism by which ALA fosters cardiovascular health benefits is usually explained in terms of it being a precursor for endogenous LC *n*-3 PUFA biosynthesis of EPA and DHA, the two key fatty acid substrates for the synthesis of eicosanoid family of biological mediators. However, in humans, the conversion of ALA to EPA and DHA is inefficient, almost negligible in the case of conversion to DHA [12,13,14]. This is due to the lack of an efficient elongation and desaturation process to convert ALA to EPA by any more than 5%–7% [12]. The primary reason for this inefficiency is explained by the *n*-6 PUFA linoleic acid (LA) competing with ALA at the level of the Δ6-desaturase enzyme complex [12,13,14], which inserts additional double bonds to these precursor fatty acids. Accordingly, the conversion of ALA to stearidonic acid (SDA, C18:4*n*-3), facilitated by this enzyme, is considered the rate-limiting step in LC *n*-3 PUFA biosynthesis in vertebrates [15].

In order to address this limited bio-conversion of ALA to EPA and beyond, there has been interest in evaluating plant oils with better conversion to EPA and DHA than ALA-rich oils. In this regard, oils containing SDA have been the subject of much interest in feeding experiments involving farm animals [16,17], aquaculture fish [18], animal models [19] and humans [20,21,22]. SDA is relatively abundant in plants of the *Boraginaceae* family. One of the commercially available non-GM sources of SDA is extracted from *Echium plantagineum* [23]. SDA has been found to be further metabolised *in vivo* and lead to increased plasma and tissue levels of LC *n*-3 PUFA both in animal models [19] and in humans [24]. For example, increased EPA and DPA have been observed following supplementation of humans with Echium oil (EO) [24]. In parallel with such compositional alterations, modification of several biochemical and physiological markers for cardiovascular disease have also been observed [25]. For example, daily supplementation with 15 g EO for four weeks lowered serum triacylglycerols in hypertriglyceridemic subjects [24], whilst a longer feeding protocol (17 g/day EO for 8-weeks equating to 2 g/day SDA) in normal and overweight individuals was accompanied by reductions in serum cholesterol, LDL-cholesterol, oxidized-LDL, HDL-cholesterol and triacylglycerols [25]. In contrast, a more recent randomized controlled trial [26] of overweight and obese subjects found no change in serum triacylglycerols following EO (1.2 g/day SDA; 6-weeks). In subjects with metabolic syndrome, further to improving plasma lipid profiles, additional benefits of EO were noted by Khunt *et al.* [25] with reductions in blood pressure and plasma insulin. Interestingly, this latter study has concluded that the collective outcomes of EO on cardiovascular risk biomarkers are broader than that exerted by fish oil (1.9 g/day EPA) itself. These benefits of EO were observed in the absence of any increase in DHA in plasma and/or peripheral blood mononuclear cells.

Biochemical studies in animals, using apoB100-only LDLrKO mice, have shown that decreased lipogenic gene expression increased intravascular lipolysis and enhanced clearance of plasma very low density lipoprotein (VLDL) as potential mechanisms for the triglyceride lowering action of EO [27,28]. In addition to lipid lowering properties, anti-atherogenic actions of EO have also been reported [29]. For example, both EO and FO were equally effective in reducing plasma triglycerides, total plasma cholesterol, VLDL and LDL-cholesterols and apoB lipoproteins, as reflected in the form of reduced aortic deposition of cholesterol, and surface lesion formation leading to retardation in atherogenesis [29]. Such collective observations from both human and animal studies have led these investigators to suggest that EO may be useful as a botanical alternative to FO in reducing hypertriglyceridemia and affording athero-protection [29,30].

Most studies with EO have reported increased accumulation of EPA and DPA with no change in DHA in blood and cell lipids [25] suggesting incomplete metabolism (elongation/desaturation) of the precursor fatty acids in EO. However, compositional data following EO supplementation on major organs (heart, kidney, liver) is lacking and it is unknown whether or not further conversion/incorporation of SDA and its metabolites has taken place, for example, in tissue specific manner. Moreover, several recent studies have reported EO is able to mimic biochemical measures of cardiovascular risk reduction benefits of FO [24,25,27,28]. Nevertheless, except for an indirect or secondary observation [19] regarding blood pressure, no datum exists in relation to any direct measure(s) of a given endpoint of cardiovascular pathophysiology that may be influenced after ingesting EO. In this context, a unique characteristic of the two major LC *n*-3 PUFA in fish oils—EPA and DHA—is their ability to modify ischemia-induced ventricular fibrillation and sudden cardiac death [31]. The anti-arrhythmic actions of EPA and DHA first demonstrated in this laboratory over two decades ago in whole animal models have now been confirmed in large scale human clinical trials [32] which has also validated the experimental model employed. To this end, despite the presence of a sound body of evidence showing increased accumulation of LC *n*-3 PUFA following dietary EO (SDA), and claimed benefits on plasma lipids, its potential to facilitate any direct anti-arrhythmic benefit has not yet been evaluated. Therefore, this study was initiated with two main objectives: (A) to compare the impact of any dose-related outcomes of EO and FO on the LC *n*-3 PUFA contents of membrane phospholipids in major tissues, and (B) to provide comparative data on the anti-arrhythmic potential of these two sources of *n*-3 PUFA, using the well-established rat model of cardiac arrhythmia, ventricular fibrillation and sudden cardiac death [33].

## 2. Materials and Methods

### 2.1. Animals, Diet and Experimental Design

A total of 126 Sprague Dawley (SD) were obtained from the Animal Resource Centre, Western Australia, at 12 weeks of age. After arrival in the Animal House, they were fed with standard rat and mouse pellets (www.specialtyfeeds.com) containing 19.6% protein, 5% fat, 4.3% crude fibre and 14.3 megajoule/kg digestible energy for a period of two weeks (acclimatisation period). The test diets were prepared by supplementing the standard rat diet (Control,) with either Echium (EO) or fish oil (FO) at three different doses (1%, 3% and 5% *w*/*w*). Hence, the total dietary fat was 5%) for the Control, and 6%, 8% or 10% for the three supplement levels, respectively. The supplemented diets were iso-caloric at any given dose-level. At the age of 14 weeks, they were randomly assigned (*n* = 18 group) to their allotted treatment groups (Control, EO-1 or FO-1, EO-3 or FO-3 and EO-5 or FO-5). The initial weight (mean ± SEM) of rats for the groups were as follows: 403 ± 8.1, 425 ± 6.2, 401 ± 12.2, 420 ± 7.0, 403 ± 7.0, 473 ± 7.3, and 400 ± 8.7 g for Control, EO-1, FO-1, EO-3, FO-3, EO-5 and FO-5, respectively. The Echium oil (Crossential 5A14) was supplied by Croda Australia (Wetherill Park, New South Wales, NSW, Australia). The fish oil used was a tuna oil high in DHA supplied by Clover Corporation (Sydney, NSW, Australia). The major *n*-3 and *n*-6 PUFA composition of the oils used are shown in Table 1. Animals were caged in groups of 4 and were provided with food and water *ad libitum* with a 12-h light-dark cycle and maintained on their allocated treatment diets for a period of 12 weeks. Body weights were recorded weekly. At the completion of pre-feeding period, the rats were subjected to coronary artery ligation as detailed below. All experimental procedures, including housing and welfare were approved by the institutional Animal Ethics Committee (CSIRO Health Sciences and Nutrition) in accordance with the Australian (National Health & Medical Research Council) code for the care and use of animals for scientific purposes.

**Table 1:** Major *n*-3 and *n*-6 composition (% total fatty acids) of Echium oil, Fish oil and Control diet used in the study.

Fatty Acid	Echium Oil (EO)	Fish Oil (FO)	Control Diet
C16:0	7.1	22.3	10.4
C16:1	-	3.0	0.21
C18:0	3.6	6.0	2.9
C18:1*n*-9	15.5	17.9	39.6
C18:2*n*-6 (LA)	15.0	1.2	27.1
C18:3*n*-6 (GLA)	11.4	0.4	-
C18:3*n*-3 (ALA)	33.1	0.9	6.3
C18:4*n*-3 (SDA)	14.2	1.6	-
C20:0	-	0.7	-
C20:1	-	2.1	0.6
C20:2	-	3.1	-
C20:4*n*-6 (ARA)	-	1.3	0.2
C20:5*n*-3 (EPA)	-	5.9	0.4
C22:5*n*-3 (DPA)	-	2.0	1.0
C22:6*n*-3 (DHA)	-	22.2	-

ALA, α-linolenic acid; ARA, arachidonic acid; DHA; docosahexaenoic acid; DPA, docosapentaenoic acid; EPA, eicosapentaenoic acid; GLA, γ-linolenic acid; LA, linoleic acid; SDA, stearidonic acid. Values represent the average of triplicate determinations.

### 2.2. Surgical Induction of Arrhythmias

The rodent arrhythmia model has been used extensively in this laboratory for many years [33]. In brief, rats were anaesthetised with a single intraperitoneal (i.p.) injection of sodium pentobarbitone (50 mg/kg). They were intubated using a tracheal tube to permit artificial ventilation upon the opening of the chest cavity. The right femoral artery was cannulated (polythene tubing, Boots Healthcare Pty Ltd, North Ryde, NSW 2113, Australia) for monitoring blood pressure. The chest wall was opened between the 2nd and 3rd ribs to permit the exteriorisation of the heart following the rupture of the myocardium. A loose ligature (Dynek Sutures, Hendon, SA 5014, Australia) was placed around the left descending coronary artery and the heart was returned to the chest cavity. Rats were allowed to stabilise for 5 min before the ligature was tightened creating acute myocardial ischemia. The ischemic period was maintained for a period of 30 min before release and further monitoring for 5 min. No attempts were made to terminate ventricular fibrillation. Blood pressure (DA 100C, Biopac Systems Inc., Goleta, CA, USA), and electrocardiogram (ECG100C, Biopac Systems Inc., Goleta, CA, USA) changes were monitored throughout the experimental period and recorded using a computer based data acquisition system (BioPak-MP100). Rats were killed by exsanguination and their hearts were placed in ice cold normal saline (0.9% NaCl). Zone at risk was assessed by the re-occlusion of the heart and the infusion of Evans Blue dye (0.5% in normal saline). The ventricles were removed and the stained area separated from the ischemic zone. The non-ischemic zone was used for fatty acid analysis. The ischemic zone was expressed as a percentage of the total ventricular weight. All animals included in the study had a % zone at risk of between 45% and 55%.

### 2.3. Assessment of Arrhythmias

Arrhythmias were assessed and classified into three types, ventricular ectopic beats (VEB), ventricular tachycardia (VT) and ventricular fibrillation (VF). The type and duration of each of these parameters (VEB, VT and VF) were assessed for each animal and a total of all arrhythmias occurring in the 30 min period of occlusion was made. Mortality was calculated as the number of deaths from VF within the group as a whole. A value of 124 s was allocated as the time for a fatal VF episode [33].

### 2.4. Tissue Collection

Following the completion of the coronary artery ligation, a blood sample of 10 mL was obtained from each animal at the abdominal aortic bifurcation. Blood samples were centrifuged (2000 *g*) for collection of plasma samples; the latter were stored at −80 °C until required. Heart (non-ischemic section), left kidney, and liver (frontal lobe) were removed from all treatment groups, cleared of any adhering tissue, blotted dry, and frozen in liquid nitrogen. Samples were then transferred to a −80 °C freezer and stored until required.

### 2.5. Fatty Acid Composition

Plasma and membrane phospholipids were extracted using chloroform-methanol [34]. The solvent layer was removed and dried under N_2_ before reconstitution in hexane. Samples were cleaned in florisil columns and eluted with 10% diethyl ether in hexane. The dried samples were dissolved in iso-octane and injected into the gas chromatagraph (GC) (Model 6890N, Agilent Technologies Australia, Mulgrave, VIC 3170, Australia). The GC was equipped with a flame ionisation detector (FID) and a BPX70 column which was 30 m long with an internal diameter of 0.53 mm, and with a 0.5 μm film thickness (SPE Analytical Service PTY Ltd, Melrose Park, NSW 2114, Australia). The temperature program was as follows: rise from 100 °C to 180 °C at 6 °C/min, and 180 °C–230 °C at 3 °C/min. Each sample involved a split injection of 2.5 μL. The carrier gas was hydrogen (30 cm/s). Fatty acids were identified by comparing their retention time to that of their respective counterparts in a standard FAME mixture (Suppleco 37, Cat. No. 47885-U, Suppelco, Belleford, PA, USA).

### 2.6. Statistics

All statistics were calculated using IBM^®^ SPSS^®^ Statistics 20 [35]. Fatty acid data in plasma and tissue, and the parametric arrhythmia data (VEB number, (VT) duration, and (VF) duration) were analysed using the SPSS General Linear Model’s Multivariate Analysis of Variance. A P-value of less than 0.05 was considered significant. For fatty acid data, oil × dose interactions were analysed in a 2 × 3 factorial omitting the Control groups. The rate of incidence of VT, VF and mortality were analysed using the Chi-Square test in SPSS. Fatty acid data were generated on a subsample of 5 animals from each group. Final number of animals per group in arrhythmia analysis ranged from 15 to 18 based on criteria for inclusion according to The Lambeth Convention [36].

## 3. Results

Dietary treatments had no apparent impact on growth and development. The final weights (mean ± SEM) of rats were 507 ± 12.2, 514 ± 11.5, 501 ± 14.7, 521 ± 10.1, 581 ± 9.9, 581 ± 9.6 and 506 ± 14.3 g for Control, EO-1, FO-1, EO-3, FO-3, EO-5 and FO-5, respectively. Average weight gain ranged from 2.06 to 2.56 g/day (*p* > 0.05) across oil types and doses.

### 3.1. Tissue Incorporations of n-3 Long-Chain Polyunsaturated Fatty Acids (LC n-3 PUFA)

Fatty acid compositions of plasma, heart, liver and kidney are presented in Table 2, Table 3, Table 4 and Table 5. Plasma samples had detectable levels of ALA that showed significant positive response to increased EO supplementation. Compared to control, plasma EPA level was significantly increased by the two higher doses of EO and by all three doses of FO (Table 2). There was also significant oil–dose interaction as the three EO doses had similar values while the FO doses exhibited significant differences in plasma EPA (FO-1 < FO-3 < FO-5). Compared to the control EO-fed rats showed higher (*p* < 0.05) accumulation of DPA in plasma whilst this LC *n*-3 PUFA was absent in FO-supplemented rats. Plasma DHA was significantly affected by both oil type and dose. It was higher on FO than EO or Control groups. Plasma from FO-1 rats had a lower percentage of DHA than FO-3 and FO-5; the difference between the latter two was not significant. The change in total plasma *n*-3 PUFA as well as EPA + DHA were largely driven by plasma DHA.

The comparative changes in the fatty acids composition of heart tissues under the different dietary treatments are shown in Table 3. Across all diet groups, neither ALA nor SDA was detected in the phospholipid fatty acids of heart tissues. The level of EPA in heart tissue was significantly affected by dose, not oil type. There was no oil–dose interaction. In contrast, the level of DPA in heart tissue was significantly affected by both oil type and dose. Percentage of DPA was significantly increased by EO but not FO. EO supplementation increased DPA levels with each increased dose, while the DPA levels across FO doses remained unchanged. The reverse was true for heart DHA. Compared to control diet, both EO and FO supplementation significantly increased total *n*-3 PUFA in heart, only FO showed a significant dose response.

Liver phospholipids did not have detectable levels of ALA or SDA (Table 4). The level of EPA in liver was affected by both oil type (FO > EO) and dose (FO-1 < (FO-3 = FO-5). There was also a significant oil–dose interaction. EPA levels were similar among Control, the three doses of EO and FO-1, whereas FO-3 and FO-5 groups had greater EPA (*p* < 0.05) than the other groups. The liver DPA concentration increased in response to EO, but not FO, supplementation. Oil type was the main determinant of liver DPA. The level of DHA in liver was significantly increased over Control by FO, but not EO, supplementation. Although there was a trend of increase in DHA with increasing dose of FO (which caused significant oil × dose interaction), the difference between FO doses was not significant. The total *n*-3 PUFA content of liver phospholipids was significantly affected by oil type and dose. Across all doses, *n*-3 PUFA levels in the liver of EO rats were similar to that of Control rats. In contrast, all FO rats had higher *n*-3 PUFA in their livers than Control or EO rats. Total *n*-3 PUFA on FO-1 was significantly lower than that observed on FO-3 and FO-5, the latter did not differ significantly. EPA + DHA values followed similar trends to that of total *n*-3 PUFA.

Kidney phospholipids did not exhibit detectable levels of ALA or SDA (Table 5). EO-3, EO-5 and all the three doses of FO had greater EPA levels than Control groups. EO-3, EO-5 and FO-1 had similar EPA levels, while FO-3 and FO-5 were the groups with the highest EPA in kidney. The level of DPA in kidney was only increased by EO, but not FO, supplementation. EO-3 and EO-5 had higher DPA than all other groups. DHA levels in kidney were similar among all EO and Control groups. In contrast, rats on the three doses of FO had significantly higher DHA in their kidney than any of the other groups. Total *n*-3 PUFA in kidney was increased by FO in a dose-related manner. Total *n*-3 PUFA in EO-1 was similar to Control groups, while EO-3 and EO-5 had higher total *n*-3 PUFA than the former.

**Figure 1:**
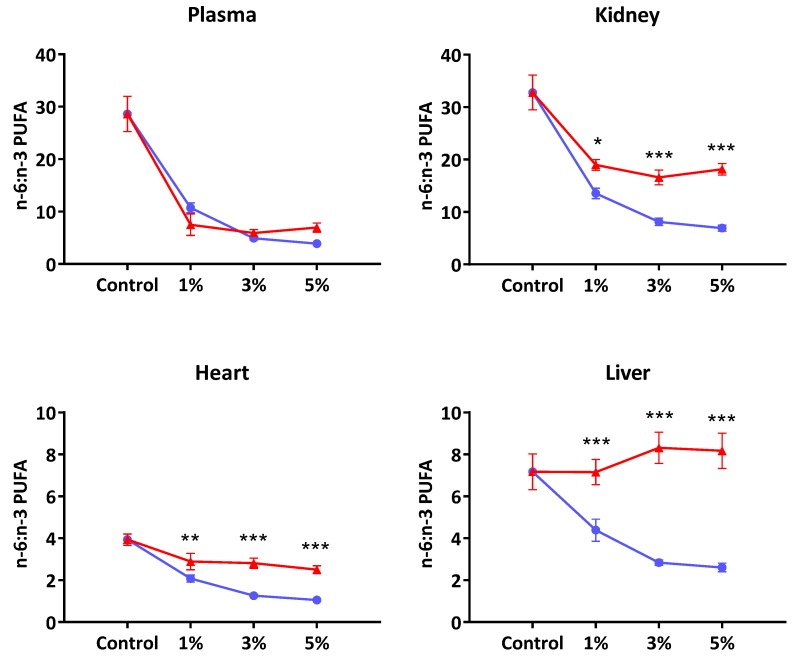
The ratio of total *n*-6 to total *n*-3 polyunsaturated fatty acids in tissue and plasma of rats fed standard laboratory diet (Control) or diet supplemented with Echium oil (EO, **red**) or fish oil (FO, **blue**) at 1, 3 and 5 percent (*w*/*w*). Values are mean ± SEM for *n* = 5 samples per group. Total *n*-3 PUFA = Sum (C18:3*n*-3, C18:4*n*-3, C20:5*n*-3, C22:5*n*-3 and C22:6*n*-3). Total *n*-6 PUFA = Sum (C18:2*n*-6, C20:2*n*-6, C20:3*n*-6 & C20:4*n*-6). *, **, and *** indicate significance at *p* < 0.05, *p* < 0.01 and *p* < 0.001, respectively.

Figure 1 presents the extent of incorporation of *n*-3 and *n*-6 PUFA (computed as total *n*-6/*n*-3 PUFA ratio) into various tissues. Except in plasma, the disparity in *n*-6/*n*-3 PUFA ratio between EO- and FO-supplemented groups increased as the oil doses increased. For example, the liver phospholipids showed virtually no change in *n*-6/*n*-3 PUFA ratio following EO whilst the FO groups showed greater incorporation of *n*-3 PUFA as the level of supplementation increased. In contrast, in the EO supplemented rats’ three organs (liver, kidney and heart), the tissue uptake of *n*-3 PUFA appeared to be plateauing much earlier at the 1% supplementation level.

**Table 2:** Plasma phospholipid fatty acid composition (% total fatty acids) of Sprague Dawley rats fed standard laboratory diet (Control) or diets supplemented with 1, 3 or 5% Echium oil (EO-1, EO-3 or EO-5) or Fish oil (FO-1, FO-3 or FO-5).

		Echium Oil			Fish Oil			Significant Effect		
Fatty Acids	Control	EO-1	EO-3	EO-5	FO-1	FO-3	FO-5	Oil	Dose	Oil × Dose
C16:0	19.4 ± 0.90	20.1 ± 0.91	18.77 ± 0.84	18.36 ± 0.66	19.12 ± 0.43	20.24 ± 0.38	20.37 ± 0.92	ns	ns	ns
C16:1*n*-9	1.9 ± 0.40 ^b^	1.1 ± 0.13 ^a^	1.2 ± 0.28 ^a^	1.0 ± 0.18 ^a^	1.4 ± 0.15 ^a^	1.7 ± 0.14 ^b^	1.90.13 ^b^	0.003	ns	ns
C18:0	14.8 ± 0.60 ^b^	12.5 ± 0.57 ^a^	12.1 ± 1.04 ^a^	13.3 ± 1.09 ^a^	14.9 ± 0.71 ^b^	14.6 ± 0.87 ^b^	15.1 ± 0.72 ^b^	0.012	ns	ns
C18:1*n*-9	17.2 ± 0.77	18.5 ± 0.29	18.0 ± 1.16	17.6 ± 0.56	18.6 ± 0.44	18.6 ± 0.57	16.9 ± 0.56	ns	ns	ns
C18:1*n*-7	5.6 ± 0.37 ^b^	4.2 ± 0.71 ^a^	3.2 ± 0.43 ^a^	3.3 ± 0.18 ^a^	5.3 ± 0.24 ^a,b^	5.1 ± 0.39 ^b^	4.7 ± 0.27 ^a,b^	<0.001	ns	ns
C18:2*n*-6 (LA)	20.4 ± 0.71 ^a,b^	21.0 ± 0.14 ^a,b^	20.0 ± 0.62 ^a,b^	19.2 ± 1.09 ^a^	21.7 ± 0.54 ^b^	19.5 ± 0.48 ^a,b^	18.3 ± 0.42 ^a^	ns	0.018	ns
C18:3*n*-3 (ALA)	0.0 ± 0.00 ^a^	1.3 ± 0.29 ^b^	2.6 ± 0.57 ^b,c^	3.1 ± 0.41 ^c^	0.0 ± 0.00 ^a^	0.0 ± 0.00 ^a^	0.0 ± 0.00 ^a^	<0.001	ns	ns
C18:4*n*-3 (SDA)	nd	nd	nd	nd	nd	nd	nd			
C20:0	2.5 ± 0.36	1.9 ± 0.82	2.1 ± 0.64	2.7 ± 0.57	1.9 ± 0.33	2.0 ± 0.40	2.5 ± 0.38	ns	ns	ns
C20:2*n*-6	nd	nd	nd	nd	nd	nd	nd			
C20:3*n*-6	0.7 ± 0.06	0.7 ± 0.02	1.0 ± 0.07	0.9 ± 0.10	0.7 ± 0.08	0.8 ± 0.09	0.6 ± 0.07	ns	ns	ns
C20:4*n*-6 (ARA)	16.2 ± 1.66 ^a,b^	16.0 ± 2.16 ^a,b^	17.6 ± 2.66 ^a,b^	17.3 ± 1.12 ^b^	13.1 ± 0.84 ^a,b^	11.1 ± 0.39 ^a^	11.8 ± 1.20 ^a,b^	0.027	ns	ns
C20:5*n*-3 (EPA)	0.2 ± 0.13 ^a^	0.7 ± 0.06 ^a,b^	1.3 ± 0.06 ^b^	1.3 ± 0.07 ^b^	0.8 ± 0.03 ^b^	2.0 ± 0.23 ^c^	2.7 ± 0.08 ^d^	<0.001	<0.001	<0.001
C24:0	nd	nd	nd	nd	nd	nd	nd			
C22:5*n*-3 (DPA)	0.0 ± 0.00 ^a^	0.5 ± 0.13 ^b^	0.9 ± 0.13 ^b^	0.8 ± 0.12 ^b^	0.0 ± 0.00 ^a^	0.0 ± 0.00 ^a^	0.0 ± 0.00 ^a^	<0.001	ns	ns
C22:6*n*-3 (DHA)	1.1 ± 0.09 ^a^	1.4 ± 0.28 ^a,b^	1.4 ± 0.11 ^a^	1.1 ± 0.15 ^a^	2.6 ± 0.28 ^b^	4.5 ± 0.26 ^c^	5.2 ± 0.53 ^c^	<0.001	0.001	<0.001
Total *n*-3 PUFA	1.3 ± 0.10 ^a^	2.7 ± 0.60 ^a,b^	3.6 ± 0.22 ^b^	3.2 ± 0.30 ^b^	3.4 ± 0.29 ^b^	6.5 ± 0.48 ^c^	7.9 ± 0.52 ^c^	<0.001	<0.001	<0.001
Total *n*-6 PUFA	37.3 ± 2.02 ^c^	18.0 ± 1.92 ^a^	21.2 ± 2.20 ^a^	21.2 ± 0.95 ^a^	35.4 ± 0.52 ^b^	31.4 ± 1.50 ^b^	30.7 ± 1.36 ^b^	<0.001	ns	0.012
EPA + DHA	1.3 ± 0.10 ^a^	2.1 ± 0.33 ^a,b^	2.7 ± 0.09 ^b^	2.4 ± 0.21 ^b^	3.4 ± 0.29 ^b^	6.5 ± 0.48 ^c^	7.9 ± 0.52 ^d^	<0.001	<0.001	<0.001
Total SFA	36.7 ± 0.64 ^b^	34.6 ± 1.22 ^a,b^	32.9 ± 1.25 ^a^	34.4 ± 1.38 ^a,b^	35.9 ± 0.60 ^b^	36.9 ± 1.23 ^b^	37.9 ± 1.98 ^b^	0.014	ns	ns
Total MUFA	24.7 ± 1.48 ^b^	23.8 ± 1.00 ^a^	22.4 ± 1.62 ^a^	22.0 ± 0.78 ^a^	25.2 ± 0.71 ^b^	25.3 ± 0.80 ^b^	23.5 ± 0.27 ^a,b^	0.027	ns	ns
Total PUFA	38.7 ± 2.00 ^a,b^	41.6 ± 2.21 ^a,b^	44.7 ± 1.91 ^b^	43.7 ± 1.25 ^a,b^	38.8 ± 0.58 ^a,b^	37.9 ± 0.97 ^a^	38.6 ± 1.86 ^a,b^	0.001	ns	ns

ARA, arachidonic acid; ALA, alpha-linolenic acid; DHA, docosahexaenoic acid; DPA, docosapentaenoic acid; EPA, eicosapentaenoic acid; LA, linoleic acid; MUFA, monounsaturated fatty acids; nd, not detected; ns, not significant (*p* > 0.05); PUFA, polyunsaturated fatty acids; SDA, stearidonic acid; SFA, saturated fatty acids. Values are mean ± SEM of *n* = 5 samples per group. Means with different superscripts are significantly different (*p* < 0.05). Total *n*-3 PUFA = Sum (C18:3*n*-3, C18:4*n*-3, C20:5*n*-3, C22:5*n*-3 and C22:6*n*-3); Total *n*-6 PUFA = Sum (C18:2*n*-6, C20:2*n*-6, C20:3*n*-6 & C20:4*n*-6); Total MUFA = Sum (C16:1*n*-9, C18:1*n*-9 & C18:1*n*-7); Total PUFA = Sum (Total *n*-3 PUFA and Total *n*-6 PUFA); Total SFA = Sum (C16:0, C18:0, C20:0 and C24:0).

**Table 3:** Heart phospholipid fatty acid composition in rats fed diets supplemented with Echium oil or Fish oil.

		Echium Oil			Fish Oil			Significant Effect		
Fatty Acids	Control	EO-1	EO-3	EO-5	FO-1	FO-3	FO-5	Oil	Dose	Oil × Dose
C16:0	13.9 ± 0.18 ^b^	13.4 ± 0.56 ^a,b^	13.2 ± 0.31 ^a,b^	12.3 ± 0.37 ^a^	15.7 ± 0.57 ^c^	15.5 ± 0.46 ^c^	15.2 ± 0.47 ^c^	<.0001	ns	ns
C16:1*n*-9	0.3 ± 0.0 ^b^	0.0 ± 0.02 ^a^	0.0 ± 0.0 ^a^	0.0 ± 0.00 ^a^	0.3 ± 0.04 ^b^	0.3 ± 0.01 ^b^	0.5 ± 0.09 ^b^	<0.001	ns	ns
C18:0	23.2 ± 0.23 ^a,b^	22.7 ± 0.44 ^a^	24.0 ± 0.47 ^b,c^	25.6 ± 0.23 ^c^	23.1 ± 0.26 ^a,b^	24.3 ± 0.35 ^c^	24.2 ± 0.36 ^c^	ns	<0.001	ns
C18:1*n*-9	6.2 ± 0.14 ^b^	5.2 ± 0.36 ^a^	4.9 ± 0.11 ^a^	4.8 ± 0.39 ^a^	5.7 ± 0.16 ^b^	4.7 ± 0.20 ^a^	4.5 ± 0.13 ^a^	ns	0.008	ns
C18:1*n*-7	5.6 ± 0.16 ^c^	4.8 ± 0.22 ^b^	4.4 ± 0.35 ^b^	3.7 ± 0.29 ^a,b^	5.4 ± 0.16 ^c^	4.4 ± 0.19 ^b^	2.9 ± 0.54 ^a^	ns	<0.001	ns
C18:2*n*-6 (LA)	21.4 ± 0.45 ^d^	19.8 ± 1.05 ^cd^	18.9 ± 0.48 ^c^	15.7 ± 0.46 ^b^	18.0 ± 0.91 ^c^	14.4 ± 0.64 ^a,b^	13.2 ± 0.40 ^a^	<0.001	<0.001	ns
C18:3*n*-3 (ALA)	nd	nd	nd	nd	nd	nd	nd			
C18:4*n*-3 (SDA)	nd	nd	nd	nd	nd	nd	nd			
C20:2*n*-6	0.2 ± 0.02 ^b^	0.0 ± 0.00 ^a^	0.0 ± 0.00 ^a^	0.0 ± 0.00 ^a^	0.2 ± 0.01 ^b^	0.1 ± 0.04 ^b^	0.1 ± 0.01 ^b^	<0.001	ns	ns
C20:3*n*-6	0.3 ± 0.02 ^a^	0.3 ± 0.03 ^a^	0.5 ± 0.04 ^b^	0.7 ± 0.04 ^c^	0.3 ± 0.02 ^a^	0.3 ± 0.03 ^a^	0.3 ± 0.02 ^a^	<0.001	<0.001	<0.001
C20:4*n*-6 (ARA)	18.1 ± 0.31 ^c^	19.0 ± 0.53 ^c^	19.5 ± 0.41 ^c^	21.2 ± 0.23 ^d^	14.9 ± 0.19 ^b^	13.4 ± 0.38 ^a^	13.4 ± 0.19 ^a^	<0.001	ns	<0.001
C20:5*n*-3 (EPA)	0.1 ± 0.03 ^a,b^	0.0 ± 0.00 ^a^	0.2 ± 0.01 ^a,b^	0.2 ± 0.06 ^b^	0.2 ± 0.02 ^a,b^	0.6 ± 0.04 ^c^	0.5 ± 0.02 ^c^	ns	0.003	ns
C24:0	0.6 ± 0.02 ^b^	0.7 ± 0.03 ^b^	0.4 ± 0.02 ^a,b^	0.6 ± 0.16 ^b^	0.4 ± 0.04 ^a^	0.3 ± 0.04 ^a^	0.2 ± 0.03 ^a^	<0.001	ns	ns
C22:5*n*-3 (DPA)	1.6 ± 0.01 ^b^	3.0 ± 0.15 ^c^	4.1 ± 0.35 ^d^	5.1 ± 0.15 ^e^	1.1 ± 0.07 ^a,b^	1.1 ± 0.04 ^a,b^	0.9 ± 0.02 ^a^	<0.001	<0.001	<0.001
C22:6*n*-3 (DHA)	8.6 ± 0.43 ^a^	11.2 ± 1.22 ^a^	9.8 ± 0.55 ^a^	10.0 ± 0.78 ^a^	14.9 ± 0.74 ^b^	20.8 ± 0.67 ^c^	24.1 ± 0.85 ^d^	<0.001	<0.001	<0.001
Total *n*-3 PUFA	10.2 ± 0.51 ^a^	14.2 ± 1.33 ^b^	14.0 ± 0.68 ^b^	15.3 ± 0.96 ^b^	16.2 ± 0.77 ^b^	22.4 ± 0.70 ^c^	25.6 ± 0.86 ^c^	<0.001	<0.001	0.001
Total *n*-6 PUFA	39.8 ± 0.58 ^c^	39.2 ± 1.07 ^c^	39.0 ± 0.77 ^c^	37.7 ± 0.39 ^c^	33.2 ± 0.89 ^b^	28.1 ± 0.61 ^a^	26.8 ± 0.58 ^a^	<0.001	<0.001	0.009
EPA + DHA	8.7 ± 0.45 ^a^	11.2 ± 1.22 ^b^	10.0 ± 0.53 ^a,b^	10.2 ± 0.82 ^a,b^	15.1 ± 0.75 ^c^	21.2 ± 0.69 ^d^	24.6 ± 0.86^e^	<0.001	<0.001	<0.001
Total SFA	37.7 ± 0.37 ^a,b^	36.7 ± 0.28 ^a^	37.7 ± 0.21 ^a,b^	38.5 ± 0.49 ^b,c^	39.2 ± 0.61 ^c^	40.1 ± 0.41 ^c^	39.7 ± 0.42 ^c^	<0.001	0.024	ns
Total MUFA	12.1 ± 0.26 ^c^	9.9 ± 0.52 ^b^	9.3 ± 0.45 ^b^	8.5 ± 0.67 ^a,b^	11.4 ± 0.25 ^c^	9.4 ± 0.35 ^b^	7.3 ± 0.64 ^a^	ns	0.001	ns
Total PUFA	50.2 ± 0.51 ^a^	53.4 ± 0.35 ^b^	53.0 ± 0.33 ^b^	53.0 ± 1.16 ^b^	49.5 ± 0.48 ^a^	50.6 ± 0.74 ^a^	52.5 ± 0.42 ^b^	0.001	ns	ns

ARA, arachidonic acid; ALA, alpha-linolenic acid; DHA, docosahexaenoic acid; DPA, docosapentaenoic acid; EPA, eicosapentaenoic acid; LA, linoleic acid; MUFA, monounsaturated fatty acids; nd, not detected; ns, not significant (*p* > 0.05); PUFA, polyunsaturated fatty acids; SDA, stearidonic acid; SFA, saturated fatty acids. Values are mean ± SEM of *n* = 5 samples per group. Means with different superscripts are significantly different (*p* < 0.05). Total *n*-3 PUFA = Sum (C18:3*n*-3, C18:4*n*-3, C20:5*n*-3, C22:5*n*-3 and C22:6*n*-3); Total *n*-6 PUFA = Sum (C18:2*n*-6, C20:2*n*-6, C20:3*n*-6 & C20:4*n*-6); Total MUFA = Sum (C16:1*n*-9, C18:1*n*-9 & C18:1*n*-7); Total PUFA = Sum (Total *n*-3 PUFA and Total *n*-6 PUFA); Total SFA = Sum (C16:0, C18:0, C20:0 and C24:0).

**Table 4:** Liver phospholipid fatty acid composition in rats fed diets supplemented with Echium oil or Fish oil.

		Echium Oil			Fish Oil			Significant Effect		
Fatty Acids	Control	EO-1	EO-3	EO-5	FO-1	FO-3	FO-5	Oil	Dose	Oil × Dose
C16:0	17.5 ± 0.71	18.5 ± 0.73	18.6 ± 0.61	18.5 ± 0.84	18.1 ± 0.20	19.0 ± 0.69	19.0 ± 0.55	ns	ns	ns
C16:1*n*-9	0.9 ± 0.14 ^b^	0.5 ± 0.09 ^a^	0.4 ± 0.06 ^a^	0.4 ± 0.06 ^a^	0.7 ± 0.09 ^b^	0.6 ± 0.04 ^a,b^	0.7 ± 0.03 ^a,b^	<0.001	ns	ns
C18:0	20.9 ± 1.42 ^a^	22.3 ± 0.40 ^a,b^	23.4 ± 0.99 ^b^	22.4 ± 0.42 ^a,b^	20.6 ± 0.75 ^a^	21.0 ± 0.69 ^a^	20.5 ± 0.42 ^a^	0.001	ns	ns
C18:1*n*-9	4.7 ± 0.27 ^a,b^	4.4 ± 0.14 ^a^	4.7 ± 0.14 ^a,b^	4.5 ± 0.30 ^a,b^	5.0 ± 0.38 ^a,b^	5.3 ± 0.26 ^a,b^	5.4 ± 0.22 ^b^	0.002	ns	ns
C18:1*n*-7	5.2 ± 0.32 ^c^	4.2 ± 0.11 ^b^	3.9 ± 0.23 ^a,b^	3.7 ± 0.13 ^a,b^	4.1 ± 0.11 ^a,b^	3.7 ± 0.17 ^a,b^	3.4 ± 0.11 ^a^	ns	0.002	ns
C18:2*n*-6 (LA)	15.0 ± 0.91 ^b^	14.5 ± 0.32 ^a,b^	13.2 ± 0.44 ^a,b^	11.9 ± 0.51 ^a^	16.6 ± 0.95 ^b^	16.1 ± 0.75 ^b^	16.2 ± 0.68 ^b^	<0.001	ns	ns
C18:3*n*-3 (ALA)	nd	nd	nd	nd	nd	nd	nd	nd		
C18:4*n*-3 (SDA)	nd	nd	nd	nd	nd	nd	nd	nd		
C20:2*n*-6	0.6 ± 0.06 ^b,c^	0.7 ± 0.04 ^c^	0.7 ± 0.02 ^c^	0.6 ± 0.04 ^b,c^	0.5 ± 0.05 ^a,b^	0.4 ± 0.04 ^a,b^	0.4 ± 0.02 ^a^	<0.001	0.006	ns
C20:3*n*-6	1.7 ± 0.15 ^a^	1.4 ± 0.09 ^a^	1.9 ± 0.08 ^a,b^	2.3 ± 0.16 ^b^	1.8 ± 0.09 ^a^	1.8 ± 0.06 ^a^	1.6 ± 0.08 ^a^	0.033	0.009	<0.001
C20:4*n*-6 (ARA)	26.9 ± 0.47 ^c^	26.9 ± 1.19 ^c^	27.7 ± 0.56 ^d^	30.0 ± 0.81 ^d^	22.6 ± 0.45 ^b^	18.9 ± 0.28 ^a^	18.5 ± 0.34 ^a^	<0.001	ns	<0.001
C20:5*n*-3 (EPA)	0.6 ± 0.08 ^a^	0.8 ± 0.16 ^a^	1.1 ± 0.10 ^a^	1.0 ± 0.14 ^a^	1.3 ± 0.13 ^a^	2.5 ± 0.36 ^b^	2.8 ± 0.35 ^b^	<0.001	0.002	0.028
C24:0	0.4 ± 0.02 ^c^	0.5 ± 0.01 ^b,c^	0.4 ± 0.01 ^b^	0.3 ± 0.09 ^a,b^	0.4 ± 0.03 ^a,b^	0.3 ± 0.02 ^a,b^	0.2 ± 0.06 ^a^	0.021	0.004	ns
C22:5*n*-3 (DPA)	0.7 ± 0.04 ^a^	1.2 ± 0.07 ^b,c^	1.2 ± 0.12 ^b,c^	1.4 ± 0.10 ^c^	0.68 ± 0.05 ^a^	1.0 ± 0.06 ^a,b^	0.8 ± 0.07 ^a^	<0.001	ns	ns
C22:6*n*-3 (DHA)	5.0 ± 0.46 ^a^	4.1 ± 0.23 ^a^	3.0 ± 0.22 ^a^	3.1 ± 0.29 ^a^	7.8 ± 0.91 ^b^	9.5 ± 0.21 ^b^	10.6 ± 0.90 ^b^	<0.001	ns	0.011
Total *n*-3 PUFA	6.3 ± 0.57 ^a^	6.1 ± 0.30 ^a^	5.3 ± 0.41 ^a^	5.5 ± 0.35 ^a^	9.7 ± 0.94 ^b^	13.0 ± 0.39 ^c^	14.2 ± 0.79 ^c^	<0.001	0.014	0.001
Total *n*-6 PUFA	43.5 ± 0.74 ^c^	42.8 ± 1.28 ^c^	42.8 ± 0.73 ^b,c^	44.3 ± 1.43 ^c^	40.9 ± 1.24 ^b,c^	36.7 ± 0.91 ^a,b^	36.3 ± 0.91 ^a^	<0.001	ns	0.031
EPA + DHA	6.3 ± 0.53 ^a^	6.1 ± 0.25 ^a^	5.3 ± 0.30 ^a^	5.5 ± 0.34 ^a^	9.7 ± 0.91 ^b^	13.0 ± 0.34 ^c^	14.2 ± 0.73 ^c^	<0.001	0.014	0.001
Total SFA	38.8 ± 0.76 ^a^	41.3 ± 1.12 ^b^	42.3 ± 1.08 ^a,b^	41.1 ± 1.26 ^a,b^	39.0 ± 0.75 ^a^	40.3 ± 1.22 ^a^	39.7 ± 0.47 ^a^	0.029	ns	ns
Total MUFA	10.8 ± 0.53 ^b^	9.8 ± 0.17 ^a^	9.0 ± 0.38 ^a^	8.5 ± 0.25 ^a^	9.8 ± 0.40 ^a,b^	9.6 ± 0.22 ^a,b^	9.5 ± 0.21 ^a,b^	0.004	ns	ns
Total PUFA	50.4 ± 0.56	49.6 ± 1.18	48.7 ± 0.92	50.4 ± 1.14	51.8 ± 0.42	50.1 ± 1.00	50.8 ± 0.30	ns	ns	ns

ARA, arachidonic acid; ALA, alpha-linolenic acid; DHA, docosahexaenoic acid; DPA, docosapentaenoic acid; EPA, eicosapentaenoic acid; LA, linoleic acid; MUFA, monounsaturated fatty acids; nd, not detected; ns, not significant (*p* > 0.05); PUFA, polyunsaturated fatty acids; SDA, stearidonic acid; SFA, saturated fatty acids. Values are mean ± SEM of *n* = 5 samples per group. Means with different superscripts are significantly different (*p* < 0.05). Total *n*-3 PUFA = Sum (C18:3*n*-3, C18:4*n*-3, C20:5*n*-3, C22:5*n*-3 and C22:6*n*-3); Total *n*-6 PUFA = Sum (C18:2*n*-6, C20:2*n*-6, C20:3*n*-6 & C20:4*n*-6); Total MUFA = Sum (C16:1*n*-9, C18:1*n*-9 & C18:1*n*-7); Total PUFA = Sum (Total *n*-3 PUFA and Total *n*-6 PUFA); Total SFA = Sum (C16:0, C18:0, C20:0 and C24:0).

**Table 5:** Kidney phospholipid fatty acid composition in rats fed diets supplemented with Echium oil or Fish oil.

	Echium Oil			Fish Oil				Significant Effect		
Fatty Acids	Control	EO-1	EO-3	EO-5	FO-1	FO-3	FO-5	Oil	Dose	Oil × Dose
C16:0	23.4 ± 0.17 ^b^	21.2 ± 0.42 ^a^	21.1 ± 0.32 ^a^	20.4 ± 0.36 ^a^	22.3 ± 0.68 ^a,b^	23.4 ± 0.22 ^b^	23.8 ± 0.37 ^b^	<0.001	ns	0.049
C16:1*n*-9	0.6 ± 0.08	0.5 ± 0.08	0.6 ± 0.09	0.4 ± 0.06	0.5 ± 0.10	0.6 ± 0.09	0.7 ± 0.03	ns	ns	ns
C18:0	21.5 ± 0.24 ^a,b^	22.8 ± 0.50 ^b^	22.7 ± 0.34 ^b^	22.8 ± 0.41 ^b^	21.3 ± 0.34 ^a,b^	21.4 ± 0.37 ^a^	21.2 ± 0.24 ^a^	<0.001	ns	ns
C18:1*n*-9	8.3 ± 0.21	8.7 ± 0.38	8.2 ± 0.67	8.6 ± 0.29	8.5 ± 0.13	8.5 ± 0.28	8.7 ± 0.16	ns	ns	ns
C18:1*n*-7	3.3 ± 0.21 ^b^	3.0 ± 0.21 ^a,b^	2.6 ± 0.28 ^a,b^	2.2 ± 0.23 ^a^	2.5 ± 0.34 ^a,b^	2.5 ± 0.21 ^a,b^	2.7 ± 0.17 ^a,b^	ns	ns	ns
C18:2*n*-6 (LA)	11.1 ± 0.34 ^b^	11.7 ± 0.26 ^b^	10.2 ± 0.21 ^a^	10.0 ± 0.24 ^a^	14.6 ± 0.28 ^d^	14.0 ± 0.21 ^c^^d^	13.4 ± 0.45 ^b,c^	<0.001	<0.001	ns
C18:3*n*-3 (ALA)	nd	nd	nd	nd	nd	nd	nd			
C18:4*n*-3 (SDA)	nd	nd	nd	nd	nd	nd	nd			
C20:0	nd	nd	nd	nd	nd	nd	nd			
C20:2*n*-6	3.0 ± 0.41 ^b^	3.6 ± 0.12 ^b^	3.7 ± 0.19 ^b^	4.0 ± 0.15 ^b^	2.0 ± 0.12 ^a^	2.6 ± 0.46 ^a^	2.3 ± 0.09 ^a^	<0.001	ns	ns
C20:3*n*-6	1.1 ± 0.07 ^a,b^	1.2 ± 0.05 ^b^	1.5 ± 0.05 ^c^	1.8 ± 0.07 ^d^	1.2 ± 0.12 ^b^	1.0 ± 0.03 ^a,b^	0.9 ± 0.04 ^a^	<0.001	ns	<0.001
C20:4*n*-6 (ARA)	24.7 ± 0.42 ^b^	23.8 ± 0.95 ^b^	25.6 ± 0.71 ^c^	26.1 ± 0.66 ^c^	22.3 ± 0.41 ^b^	19.9 ± 0.83 ^a,b^	19.6 ± 0.40 ^a^	<0.001	ns	0.003
C20:5*n*-3 (EPA)	0.0 ± 0.00 ^a^	0.5 ± 0.04 ^a^	0.6 ± 0.05 ^b^	0.7 ± 0.05 ^b^	1.0 ± 0.11 ^b^	2.1 ± 0.26 ^c^	2.5 ± 0.16 ^c^	<0.001	<0.001	<0.001
C24:0	1.9 ± 0.05 ^c^	1.7 ± 0.01 ^a,b,c^	1.7 ± 0.09 ^a,b^	1.6 ± 0.05 ^a^	1.9 ± 0.03 ^b^	1.9 ± 0.04 ^b^	1.8 ± 0.05 ^a,b,c^	0.001	ns	ns
C22:5*n*-3 (DPA)	0.0 ± 0.00 ^a^	0.3 ± 0.01 ^b^	0.5 ± 0.03 ^c^	0.4 ± 0.11 ^c^	0.0 ± 0.00 ^a^	0.0 ± 0.00 ^a^	0.0 ± 0.00 ^a^	<0.001	ns	ns
C22:6*n*-3 (DHA)	1.2 ± 0.12 ^a^	1.2 ± 0.06 ^a^	1.2 ± 0.10 ^a^	1.0 ± 0.09 ^a^	1.9 ± 0.13 ^b^	2.3 ± 0.18 ^b,c^	2.5 ± 0.20 ^c^	<0.001	ns	0.015
Total *n*-3 PUFA	1.2 ± 0.12 ^a^	2.0 ± 0.10 ^a^	2.3 ± 0.16 ^b^	2.1 ± 0.14 ^b^	2.9 ± 0.20 ^b^	4.4 ± 0.34 ^c^	5.0 ± 0.32 ^d^	<0.001	<0.001	0.001
Total *n*-6 PUFA	36.8 ± 0.75 ^b^	36.7 ± 0.72 ^b^	37.3 ± 0.70 ^b^	37.8 ± 0.42 ^b^	38.1 ± 0.70 ^b^	34.9 ± 0.84 ^a^	33.9 ± 0.32 ^a^	0.005	0.045	0.001
EPA + DHA	1.2 ± 0.12 ^a^	1.7 ± 0.09 ^a^	1.8 ± 0.14 ^a^	1.7 ± 0.08 ^a^	2.9 ± 0.20 ^b^	4.4 ± 0.34 ^c^	5.0 ± 0.32 ^c^	<0.001	<0.001	<0.001
Total SFA	46.8 ± 0.21 ^b^	45.7 ± 0.59 ^a,b^	45.5 ± 0.38 ^a,b^	44.8 ± 0.30 ^a^	45.5 ± 0.45 ^a,b^	46.7 ± 0.62 ^b^	46.8 ± 0.25 ^b^	0.012	ns	ns
Total MUFA	12.2 ± 0.35	12.2 ± 0.25	11.3 ± 0.82	11.2 ± 0.45	11.5 ± 0.55	11.5 ± 0.40	12.1 ± 0.34	ns	ns	ns
Total PUFA	41.0 ± 0.46 ^a^	42.2 ± 0.81 ^a,b^	43.2 ± 0.69 ^a,b^	44.0 ± 0.46 ^b^	43.0 ± 0.76 ^a,b^	41.8 ± 0.66 ^a,b^	41.0 ± 0.35 ^a^	0.044	ns	0.028

ARA, arachidonic acid; ALA, alpha-linolenic acid; DHA, docosahexaenoic acid; DPA, docosapentaenoic acid; EPA, eicosapentaenoic acid; LA, linoleic acid; MUFA, monounsaturated fatty acids; nd, not detected; ns, not significant (*p* > 0.05); PUFA, polyunsaturated fatty acids; SDA, stearidonic acid; SFA, saturated fatty acids. Values are mean ± SEM of *n* = 5 samples per group. Means with different superscripts are significantly different (*p* < 0.05). Total *n*-3 PUFA = Sum (C18:3*n*-3, C18:4*n*-3, C20:5*n*-3, C22:5*n*-3 and C22:6*n*-3); Total *n*-6 PUFA = Sum (C18:2*n*-6, C20:2*n*-6, C20:3*n*-6 & C20:4*n*-6); Total MUFA = Sum (C16:1*n*-9, C18:1*n*-9 & C18:1*n*-7); Total PUFA = Sum (Total *n*-3 PUFA and Total *n*-6 PUFA); Total SFA = Sum (C16:0, C18:0, C20:0 and C24:0).

### 3.2. Arrhythmia Risk

**Figure 2:**
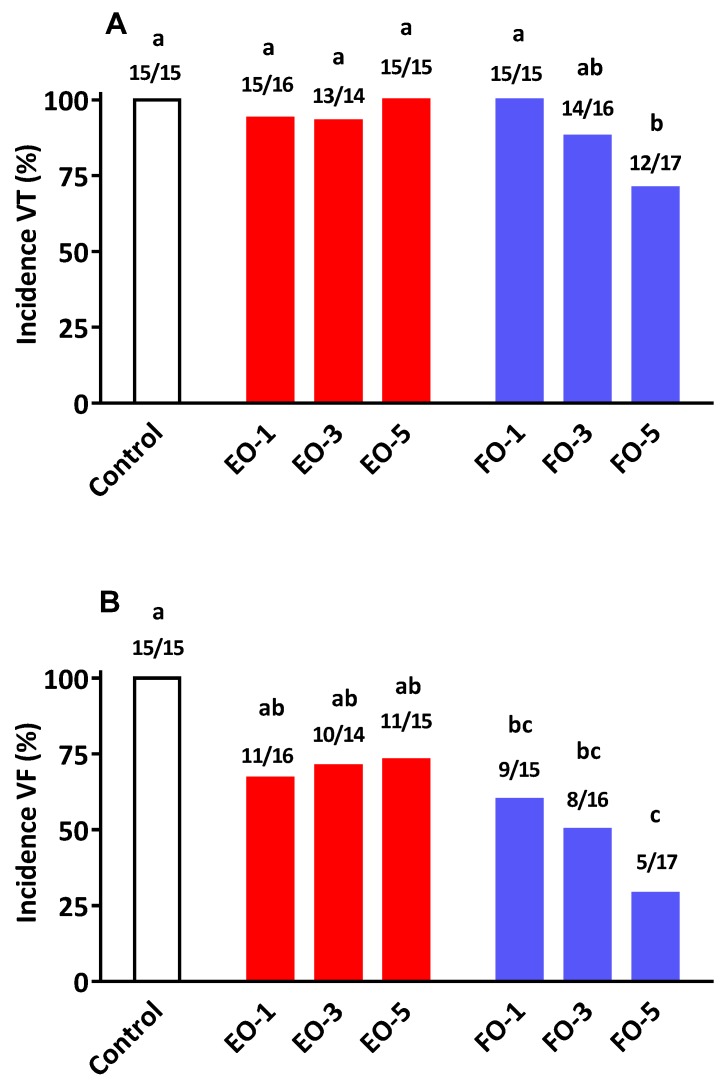
Percentage of incidence of (**A**) ventricular tachycardia (VT) and (**B**) ventricular fibrillation (VF) in rats fed standard laboratory diet (Control) or diet supplemented with Echium oil (EO, **red**) or Fish oil (FO, **blue**) at 1, 3 and 5 percentage (*w*/*w*). Numbers on top of the bars represent the incidence of VT or VF that occurred over the total number of animals measured. ^a, b, c^ Bars with different letters are significantly different (*p* < 0.05).

The results on various arrhythmia parameters are presented in Table 6 and Figure 2. The % incidence of VT was similar (nearly 100%) for Control, EO-1, EO-3, EO-5, FO-1 and FO-3 (Figure 2A). VT in FO-5 rats was lower than that observed under all other groups except FO-3 (*p* < 0.05). Across doses, the average % incidence under FO and EO oils were 86% and 96%, respectively (*p* = 0.114). There was a significant oil–dose interaction (*p* = 0.047) for VT incidence. There was also significant oil by dose interaction (*p* = 0.031) for VT duration. There was a gradual decline in VT duration with increasing FO supplementation. This trend was not evident across EO doses (Table 6).

There was a marked decline in % VF incidence as the dose of FO increased (*p* < 0.01; Figure 2B). Across doses, there was a significant oil type effect, with the average incidence rates under FO and EO oils being 46.3% and 71.3%, respectively (*p* = 0.016). As shown in Figure 2B, there was an oil–dose interaction (*p* < 0.05); whereas % incidence of VF remained similar across all the three doses of EO, it decreased with increasing doses of FO. The changes observed in duration of VF with increasing oil dose were markedly different between the two oils. In FO rats, duration of VF decreased with each increasing dose of FO (Table 6). The oil–dose interaction was also significant for VF duration (*p* < 0.05). FO feeding was associated with greater cardio-protection than that observed under EO feeding. Percentage of mortality in EO-3 groups was similar to that observed in FO fed rats.

**Table 6:** The effects of oil type and dose on parameters of cardiac arrhythmia in Sprague Dawley rats fed diets supplemented with Echium oil (EO) or fish oil (FO) following coronary artery ligation.

	Dietary Groups						
	Control	EO-1	EO-3	EO-5	FO-1	FO-3	FO-5
VEB (Total, *n*)	821 ± 161	596 ± 197	679 ± 156	556 ± 191	723 ± 210	490 ± 137	365 ± 114
VT duration (s)	45.1 ± 10 ^b^	30.4 ± 12.9 ^b^	37.5 ± 12.6 ^b^	33.2 ± 13.7 ^b^	35.6 ± 13.2 ^b^	29.0 ± 12.0 ^b^	18.2 ± 9.0 ^a^
VF duration (s)	94.4 ± 15.5 ^c^	69.8 ± 18.7 ^b^	24.2 ± 12.1 ^a^	75.8 ± 17.1 ^b^	51.5 ± 20.2 ^b^	33.9 ± 12.3 ^a,b^	22.7 ± 12.0 ^a^
Total mortality (%)	69 (9/15)	37.5 (6/16)	14.3 (2/14)	40 (6/15)	20 (3/15)	12.5 (2/16)	17.6 (3/17)

Values are mean ± SEM. Treatment diet groupings are similar to that described in Table 2. Sample size: number per group was 18 animals. Ischemic period maintained for 30 min. Numbers in parenthesis show actual numbers representing the % death. VEB, VT and VF defined as ventricular ectopic beats, ventricular tachycardia and ventricular fibrillation, respectively. Within row, means with different superscripts are significantly different (*p* < 0.05).

## 4. Discussion

The present study addressed two key questions relating to tissue fatty acid composition and cardiac arrhythmia outcomes following dietary supplementation with oils rich in *n*-3 PUFA of different origin. EO contained *n*-3 PUFA of C = 18 length (29% ALA and 14% of SDA) compared to FO which had a total LC *n*-3 PUFA, C ≥ 20 (EPA + DHA) of >30%. Both oil types resulted in marked differences in the composition of membrane phospholipids of various tissues. The previously reported [37] anti-arrhythmic actions of FO were reconfirmed in the present study. Whilst EO also led to an increased accumulation of LC *n*-3 PUFA, mainly via EPA and DPA, the extent of protection against ischemia-induced cardiac arrhythmia and sudden cardiac death was less than that observed at comparable levels of FO supplementation.

The fatty acid data from this study suggested that ALA and SDA were virtually converted to longer chain *n*-3 PUFA in heart and liver phospholipids, with no trace of 18C *n*-3 PUFA left. Previous studies have also shown very efficient conversion of both ALA and SDA to EPA and DPA in the rat liver [19]. Overall, the pattern of changes in EPA, DPA and DHA in plasma and tissue were consistent with reports from previous rat [19] and human studies [20]. The first salient feature of this study was that SDA supplementation did not yield nutritionally/physiologically meaningful DHA levels in plasma or tissue. In almost all published data, the magnitude of changes in tissue or plasma DHA as a consequence of supplementation with oils containing 18C *n*-3 PUFA were usually negligible [12]. On the contrary, SDA feeding has consistently shown significantly increased levels of EPA and DPA in plasma and tissues [19]. Our data provides further evidence that in rats there is efficient conversion of SDA up to DPA, but not DHA. In this regard, these findings also mimic the observations in humans with SDA-rich oils [20,21,22]. For example, James *et al.* [20] who compared the conversion of ALA, SDA to ≥20C *n*-3 PUFA (EPA, DPA and DHA) reported that dietary SDA led to an increase in EPA and DPA concentrations but not DHA levels in plasma and/or erythrocytes in a double blind, parallel group design study of six weeks’ duration. The efficiency of increasing tissue EPA was 1.0, 0.3 and 0.07 for EPA, SDA and ALA, respectively. Similarly, Krul *et al.* [38] showed that the efficiency of apparent conversion of SDA to EPA in human RBC was 41%, 26% and 17% of dietary SDA for doses of 0.61, 1.89 and 5.32 g/day.

Studies using 18C *n*-3 oils have shown that the efficiency of conversion of 18C *n*-3 PUFA to ≥20C *n*-3 PUFA declines as the dose (en%) of the dietary 18C *n*-3 PUFA is increased. Gibson *et al.* [39] compared 54 different diet combinations, including various ratios of *n*-6 polyunsaturated linoleic acid (LA) to ALA (*n*-3 PUFA) to determine DHA synthesis from ALA in rats. They showed plasma phospholipid EPA, DPA and DHA increased rapidly within a narrow range—between 0 and 2 en% of dietary ALA, but suppressed to basal levels (2% total fatty acids) when the en% derived from total PUFA (LA + ALA) reached 3 en% and above.

Taken collectively, the fatty acid changes in plasma and tissues following SDA feeding had two main outcomes: increases in EPA and DPA and no change in the basal levels of DHA. These findings mirror what other studies have shown to be the case for plant-based C18 omega-3 oils in humans and different animal species (see review by Brenna *et al.* [12]). Furthermore, the present study has clearly shown that the patterns of changes in LC *n*-3 PUFA in plasma and tissues are markedly different between EO and FO. In FO-supplemented rats, EPA and DHA generally increased with greater availability of LC *n*-3 PUFA. For most parts the increased LC PUFA in plasma and tissue following supplementation with FO can be explained by way of direct incorporation of dietary EPA and DHA with some elongation and desaturation of EPA taking place. In contrast, any increase in LC *n*-3 PUFA following dietary EO would be due to further metabolism of the two C18 *n*-3 precursor fatty acids ALA and SDA since pre-formed C20 fatty acids were absent in the diet. In EO-supplemented rats, only cardiac muscle phospholipids showed a dose-related increase in DPA. In all other cases, the changes in tissue and plasma EPA and DPA beyond the first dose (EO-1) were minimal, suggesting that the linear response phase may be between 0% and 1% dietary Echium oil.

Although the tissue levels of EPA and DHA in the FO fed rats tended to show a dose-related uptake, there exist clear tissue specific differences with regard to the extent of incorporation and further metabolism of these two fatty acids. For example, compared to DHA, the EPA content of cardiac muscle showed only a minor increase amounting to <0.5% of total phospholipid fatty acids even at the 5% oil supplementation level. This differed markedly with DHA where a clear dose-related accumulation was observed (Table 3), and accounted for nearly 24% of cardiac membrane fatty acids at the highest dose tested (5% *w*/*w*). It is also evident that this increase in *n*-3 PUFAs has occurred primarily at the expense of the two major *n*-6 PUFA; LA (18:2*n*-6) and AA (20:4*n*-6). Compared to the control group, the displacement of LA by *n*-3PUFA amounted to 16%, 33% and 35% at the three dose levels of FO, respectively. AA displacement amounted to 25% at the 5% supplementation level. In contrast, the LA levels in the plasma, liver and kidney had all remained unaffected despite an increased presence of dietary *n*-3 PUFA. Similarly, the AA content of liver and kidney tended to show more resistance to be displaced by greater availability of dietary *n*-3 PUFA. Taken collectively, these observations would lend further support to the role membrane phospholipids play in maintaining the physiological functioning of specific tissues and organs. As previous publications [40,41,42,43,44] showed the type and amount of fatty acids in cell membranes not only influence the physical properties of the membrane bilayer (e.g., fluidity, lipid micro domains/rafts), but also modulate important biochemical functions—ion channels, transporters and enzymes. In addition, membrane and intracellular fatty acids provide substrates for the synthesis of numerous biochemical mediators including eicosanoid family of autacoids.

It is noted that in the three organs studied—liver, kidney and heart—the extent of perturbation of fatty acid composition by EO was much less, and reached saturation at the 1% level, compared to the changes observed following feeding FO rich in EPA and DHA that appeared to be dose-related (Figure 1). However, the possibility exists for the relatively high presence of *n*-6 PUFA (26%) as well as ALA (33%) in the EO to interfere with further metabolism of SDA which was present at a much lower level (14%). It is more likely that any substrate competition would be due to LA rather than ALA since the genetically modified SDA-soybean preparation which contained lower ALA than SDA levels [21] also led to similar compositional changes as observed in the present study. The LA content in SDA-soybean was 31% compared to 16.6% SDA.

The anti-arrhythmic actions of *n*-3 PUFA has been attributed to favourable changes to the heart membrane structure, favourable modulation of ion channels (e.g., Ca^2+^) in cardiac tissue, and improved myocardial oxygen efficiency [31]. The present results conclude that while there is some anti-arrhythmic action arising from EO consumption, the efficacy is not equivalent to that achieved with FO supplementation. For example, increasing the level of dietary EO neither arrested the development of VT nor its deterioration into the more serious condition of VF (Figure 2). This differed markedly with that observed following the provision of FO, where the progression of VT to VF was reduced in a dose-related manner. At the highest supplementation level (5% *w*/*w*) FO not only reduced the incidence of VF (29% *vs.* 73% in EO group) but such episodes once occurred lasted a much shorter period of time compared to EO and/or the control group (Table 6). It is of interest to note that at the 3% supplementation level of EO displayed certain cardioprotective qualities (VF duration and % mortality) comparable to those found with FO. The reason(s) for this apparent protective actions of EO, only at this particular dose level, is difficult to explain since such benefits were not repeated at the higher supplementation level of 5%. Tissue fatty acid compositional data showed no clear differences between 3% and 5% oil incorporation levels. Furthermore, it is highly unlikely that a concentration–effect relationship for cardioprotection follows a bell-shape curve within the narrow band of EO feeding used in this study. Collectively, our findings in relation to cardio-protection and tissue fatty acid compositional alterations mirror previous studies where direct comparisons between SDA and EPA have been made with respect to several other biomarkers of cardiovascular health [20,21,25].

The data from this study and those published by others indicate that SDA containing oils lead to elevated plasma and tissue LC *n*-3 PUFA. However, the magnitude of changes reported from this study and that from the literature does not provide evidence of equivalence in potency between SDA-containing oils and fish oils. In addition, potential anti-arrhythmic action of SDA oils needs to be investigated further using other animal models as well as using pure SDA or more enriched supplements to minimise the confounding effect of ALA that is present in commercial sources of SDA oils.

In summary, evaluation of the anti-arrhythmic actions of EO and FO in a rat arrhythmia model is a novel contribution from this study. We conclude that feeding Echium oil favourably changes the *n*-3 PUFA profiles of blood and tissues in rats, especially with respect to DPA in heart tissue. Although there are emerging evidence to suggest DPA may possess unique physiological actions including anti-platelet aggregation, pancreatic lipase inhibition and potential anti-obesity effects, its efficacy in affording direct cardio-protection has not been evaluated to date using pre-formed and more pure forms of the fatty acid. The present study provides some indirect evidence for a potential role for DPA since the changes in *n*-3 PUFA profile following EO were associated with some anti-arrhythmic action, although the extent of protection did not match that achieved by FO at all three dose levels studied. Furthermore, the increased DPA may play other beneficial roles unrelated to cardiac arrhythmia. This needs to be further investigated in animal models and substantiated in relevant human cohorts.
